# COVID–19-associated coagulopathy: An exploration of mechanisms

**DOI:** 10.1177/1358863X20932640

**Published:** 2020-06-19

**Authors:** Meaghan E Colling, Yogendra Kanthi

**Affiliations:** 1 Division of Intramural Research, National Heart, Lung, and Blood Institute, National Institutes of Health, Bethesda, MD, USA; 2 Department of Internal Medicine, Division of Cardiovascular Medicine, University of Michigan, and Ann Arbor Veterans Administration Healthcare System, Ann Arbor, MI, USA

**Keywords:** anticoagulation, antiplatelet, COVID-19, inflammation, neutrophils, thrombosis, vascular endothelium, venous thromboembolism (VTE)

## Abstract

An ongoing global pandemic of viral pneumonia (coronavirus disease [COVID-19]), due to the virus SARS-CoV-2, has infected millions of people and remains a threat to many more. Most critically ill patients have respiratory failure and there is an international effort to understand mechanisms and predictors of disease severity. Coagulopathy, characterized by elevations in D-dimer and fibrin(ogen) degradation products (FDPs), is associated with critical illness and mortality in patients with COVID-19. Furthermore, increasing reports of microvascular and macrovascular thrombi suggest that hemostatic imbalances may contribute to the pathophysiology of SARS-CoV-2 infection. We review the laboratory and clinical findings of patients with COVID–19-associated coagulopathy, and prior studies of hemostasis in other viral infections and acute respiratory distress syndrome. We hypothesize that an imbalance between coagulation and inflammation may result in a hypercoagulable state. Although thrombosis initiated by the innate immune system is hypothesized to limit SARS-CoV-2 dissemination, aberrant activation of this system can cause endothelial injury resulting in loss of thromboprotective mechanisms, excess thrombin generation, and dysregulation of fibrinolysis and thrombosis. The role various components including neutrophils, neutrophil extracellular traps, activated platelets, microparticles, clotting factors, inflammatory cytokines, and complement play in this process remains an area of active investigation and ongoing clinical trials target these different pathways in COVID-19.

## Introduction

In December 2019, a new betacoronavirus (severe acute respiratory syndrome coronavirus 2 [SARS-CoV-2]), thought to originate in Wuhan, China, emerged as a novel human pathogen for viral pneumonia (coronavirus disease [COVID-19]), resulting in an ongoing pandemic.^1,2^ The number of cases worldwide now exceeds five million, with more than 350,000 associated deaths, triggering a global effort to understand the predictors of disease severity for rapid triage, and the pathology of disease for rational therapeutic development and clinical trials. A consistent finding in early case series in China and New York City is an association between elevations in D-dimer and fibrin(ogen) degradation products (FDPs) and increasing COVID-19 severity and mortality.^3–7^ We aim to review the available data on the coagulopathy observed in COVID-19 and draw from studies of prior viral epidemics to explore possible mechanisms and therapies.

Coronaviruses are enveloped, non-segmented, positive-sense RNA viruses of the *Nidovirales* order within the *Coronaviridae* family. Different strains are infectious to a broad range of animals including humans, bats, cats, racoon dogs, rabbits, pigs, and cattle.^8^ In general, coronavirus infections in humans are mild; however, two recent epidemics of betacoronaviruses – SARS in 2003^9–11^ and Middle East Respiratory Syndrome (MERS) in 2012^12,13^ – were associated with significant mortality with death rates around 10% and 35%, respectively.^14,15^ While the observed case fatality rate for the COVID-19 pandemic is lower,^16,17^ the population at risk is much higher due to the global spread of the disease and the infectivity of the virus,^18^ and worldwide fatalities already exceed those in the prior epidemics.

Common clinical manifestations of patients with COVID-19 include fever and cough, and less commonly fatigue, dyspnea, headache, sore throat, anosmia, nausea, vomiting, or diarrhea.^6^ In the largest case series to date of over 44,000 patients with COVID-19, > 75% of cases were mild, 14% were severe, and 5% were critical, with an overall case fatality rate of 2–2.5%. All deaths occurred in patients with critical disease (in which the case fatality rate was almost 50%).^19^ While the majority of critically ill patients with COVID-19 have isolated respiratory failure, often acute respiratory distress syndrome (ARDS), multiple organ dysfunction occurs in 20–30% of patients with critical illness and more often in fatal cases.^16^ Hematologic findings, such as mild to moderate thrombocytopenia and lymphopenia, are associated with COVID-19;^20,21^ however, the most significant and concerning vascular aspect of this disease is coagulopathy. We have attempted to summarize the data on the pathogenesis, epidemiology and outcomes related to COVID-19-coagulopathy and thrombotic disease using PubMed as well as the pre-print server https://medrxiv.org (date of last search April 23, 2020).

## Coagulopathy of SARS-CoV-2 and other infections

There is particular interest in the coagulopathy in patients with COVID-19 as abnormal coagulation parameters, most consistently elevations in D-dimer and FDPs, are associated with disease severity.^22,23^ An elevated D-dimer, the most common coagulation abnormality in COVID-19 (found in up to 45% of patients), is an independent risk factor for death,^6,22,24,25^ and patients with D-dimer greater than 1000 ng/mL are almost 20 times more likely to die from their infection than patients with lower D-dimer values.^25^ In contrast, most patients with COVID-19 have a normal or mildly prolonged prothrombin time (PT) and a normal or shortened activated partial thromboplastin time (aPTT) on presentation and these labs are not reliably associated with disease severity.^5,17,22,24,25^ Both initial and longitudinal monitoring of coagulation parameters can predict disease severity, as elevated D-dimer and FDP levels on admission and decreased levels of fibrinogen and antithrombin III during the admission are associated with death.^23^ Although changes in plasminogen activator inhibitor-1 (PAI-1) levels and activity have not been studied, an increase in the PAI-1/tissue plasminogen activator (t-PA) ratio would not be unexpected. These findings may be due to uncontrolled activation of coagulation with ongoing consumption and widespread microvascular thrombosis.

While early descriptions of the coagulopathy identified it as disseminated intravascular coagulation (DIC), in DIC, unlike in severe COVID-19, platelet count and PT prolongation correlate with sepsis severity and mortality, while fibrinogen and FDPs levels do not.^26,27^ And while the majority of patients who die from COVID-19 develop some laboratory evidence of DIC during their admission, elevations in D-dimer and prolonged PT with mild thrombocytopenia and normal fibrinogen are commonly seen.^23^ Thromboelastography in patients with COVID-19 in the ICU shows a hypercoagulable state.^28^ These observations suggest the underlying pathophysiology in at least a subset of critically ill patients with COVID-19 is distinct from traditional systemic DIC and may be due to a unique coagulopathy.

Elevations in D-dimer are common in critical illness and are associated with disease severity and mortality in many severe infections.^29–31^ Patients with influenza, SARS, HIV, hantavirus, Ebola virus, and dengue have elevations in D-dimer, prothrombin fragments, thrombin–antithrombin complexes, and/or plasmin-α_2_-antiplasmin complexes.^32^ Similar to patients with SARS-CoV-2 infections, there is an association between elevated D-dimer and mortality in patients with H1N1 and H5N1, which is not seen in SARS.^33–35^

Additionally, in the H1N1 pandemic, patients with severe disease had high rates of venous thromboembolism (VTE) and many patients with thromboembolism did not have evidence of systemic DIC.^36–39^ Patients with ARDS from H1N1 infection had a greater than 20-fold increase in risk of pulmonary embolism compared to patients with ARDS unrelated to H1N1.^39^ Empiric therapeutic anticoagulation in patients with ARDS was associated with decreased rates of VTE in patients with ARDS from H1N1, but had no effect on VTE rates in patients with ARDS unrelated to H1N1 infection. There are reports of VTE in patients with COVID-19, despite concerns regarding underdiagnosis given baseline elevations in D-dimer, as well as pragmatic challenges in diagnostic imaging while in isolation, including use of personal protective equipment and longer duration of exposure of health care workers.^40,41^ Although data remain scarce, there are increasing reports of arterial thrombotic events including ischemic strokes in patients with COVID-19.^41–43^ Myocardial injury, defined by elevations in cardiac troponin levels, is common in patients hospitalized with COVID-19 and is associated with severe disease and high risk of mortality.^44,45^ Myocardial injury may result from systemic inflammatory response syndrome (SIRS) and inflammation as well as due to acute thrombotic events.^46,47^ Similar observations of myocardial injury have been found in patients with other viral infections.^48,49^

## Pathologic findings in SARS-CoV-2 infection

**Figure 1.**
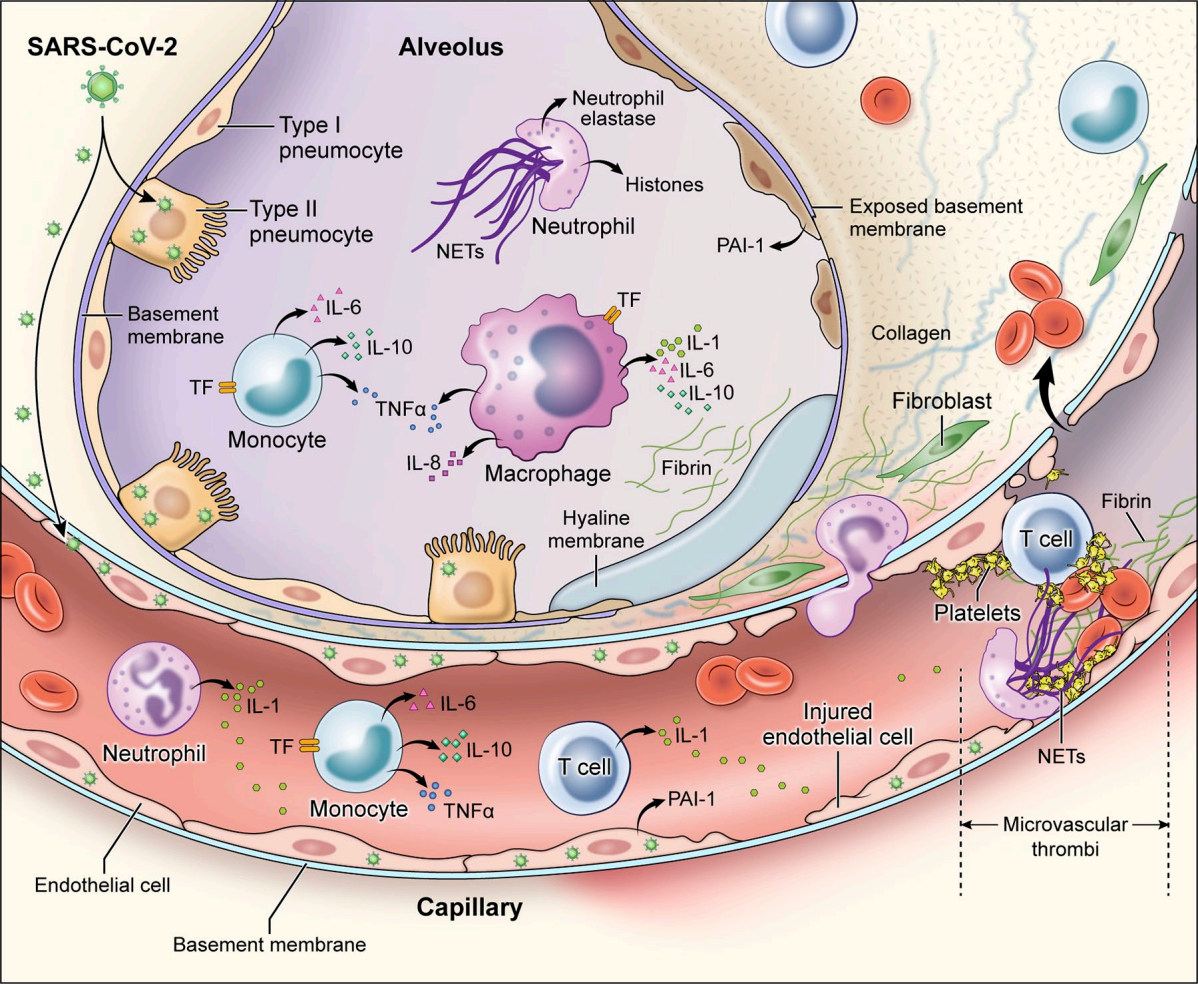
Immune activation and mechanisms of coagulopathy in patients with coronavirus disease 2019 (COVID-19). Multiple processes may contribute to COVID-19-associated coagulopathy including direct infection of type II pneumocytes and endothelial cells, leading to barrier dysfunction and increased permeability; inflammatory responses characterized by activation of T cells, neutrophils, monocytes, macrophages, and platelets resulting in exuberant inflammatory cytokine release (including IL-1, IL-6, IL-10, TNF-α), monocyte-derived TF and PAI-1 expression; and culminating in the development of microvascular and macrovascular thrombi composed of fibrin, NETs, and platelets. IL, interleukin; NETs, neutrophil extracellular traps; PAI-1, plasminogen activator inhibitor-1; TF, tissue factor; TNF-α, tumor necrosis factor-alpha.

Although there are only a few published pathologic reports of patients with COVID-19, histopathology of lung specimens from patients with early disease shows characteristic findings of ARDS and evidence of small vessel occlusion.^50,51^ There are several mechanisms by which SARS-CoV-2 infection may result in microvascular and macrovascular thrombosis, including cytokine storm with activation of leukocytes, endothelium and platelets resulting in upregulation of tissue factor, activation of coagulation, thrombin generation and fibrin formation,^52^ deranged coagulation with imbalances in PAI-1, tissue factor pathway inhibitor, and activated protein C that promotes fibrin generation and limits fibrinolysis,^53,54^ hypoxic vaso-occlusion, and direct viral effects with cell activation (Figure 1). It remains an active area of investigation whether these are specific to SARS-CoV-2 infection or a final common pathway in the thromboinflammatory response to viral infections and a marker of disease severity. Early COVID-19 autopsy reports have also identified a possible role for neutrophils as microvascular thrombi contained numerous neutrophils, which in some cases were partially degenerated, consistent with neutrophil extracellular traps (NETs).^55,56^ NETs are tangles of DNA released from neutrophils, and are decorated with antimicrobial and nuclear proteins that propagate intravascular thrombosis.^57,58^ NETs initiate both the extrinsic and contact pathways by augmenting presentation of tissue factor, activation of factor XII (FXII), as well as trapping and activating platelets.^59–62^ Consistent with these observations, patients with severe COVID-19 have elevated serum markers of neutrophil activation and NET formation.^63^ In one study, neutrophil activation measured in serum correlated with, and sometimes preceded, VTE in patients with COVID-19.^64^

## Dysregulation of hemostasis and coagulopathy in acute respiratory distress syndrome (ARDS)

Thrombi in the pulmonary micro- and macrovasculature are observed in patients with ARDS with or without overt DIC, and changes consistent with a prothrombotic state have been found both in blood and in alveolar fluid studies of these patients.^65,66^ Higher levels of FDPs and D-dimer are seen in patients who developed ARDS as compared to patients with similar predisposing conditions that did not develop ARDS.^67^ Lower levels of protein C and higher levels of soluble thrombomodulin and PAI-1 are also associated with multiple organ failure, disease severity, and mortality in ARDS in some studies.^53,68–72^ Finally, plasma and alveolar levels of tissue factor are higher in patients with ARDS than patients with pulmonary edema.^73^ Mechanistically, there is increased thrombin generation by tissue factor coupled with an impaired fibrinolytic response due to elevations in PAI-1. Elevations in D-dimer, a breakdown product of crosslinked fibrin, may result from residual t-PA/plasmin activity, as well as from alternative fibrinolytic pathways such as human neutrophil elastase activity.^74,75^

As patients with COVID-19 frequently have isolated pulmonary findings, the initial hemostatic dysregulation may be localized to the lungs as a consequence of the bidirectional relationship between the innate immune system and thrombosis. Activated platelets through degranulation and coordinated interactions with monocytes, dendritic cells, and neutrophils, as well as activated T cells, NETs, tissue factor-bearing microparticles, and coagulation proteases may facilitate this crosstalk.^54,76,77^ In this model, immune cells, inflammatory cytokines, and pathogen-associated molecular patterns induce thrombi consisting of fibrin, monocytes, neutrophils, and platelets.^57,58,78^ These immunothrombi initially serve a protective purpose, promoting pathogen recognition and creating a sterile barrier against further pathogen invasion, but can become maladaptive and injurious to tissue and organ perfusion.^57,79,80^ During this process, there is abundant intra- and extra-vascular fibrin deposition and impaired fibrinolysis, which has been well described in ARDS.^81,82^ In postmortem studies, both macro- and microvascular thrombi are common in patients in ARDS (observed in up to 95% of patients).^82,83^ In COVID-19, the alveolar immunothrombotic response may be an attempt to limit dissemination of SARS-CoV-2 outside the alveoli.

Findings from the SARS epidemic provide possible viral-specific mechanisms for ARDS and uncontrolled coagulation. Autopsy studies of patients who died of SARS pneumonia, identified the SARS-CoV spike (S) protein in cells expressing the receptor angiotensin-converting enzyme 2 (ACE2),^84–87^ the leading candidate receptor for SARS-CoV-2.^88,89^ Binding of the S protein to ACE2 induces expression of a nuclear factor kappa B (NFκB)-driven inflammatory module, resulting in production of proinflammatory cytokines including monocyte chemoattractant protein 1 (MCP-1), transforming growth factor-beta 1 (TGF-β1), tumor necrosis factor-alpha (TNF-α), interleukin (IL)-1β, and IL-6, which have been implicated in thrombogenesis.^90^ Although inflammatory responses are important in host-defense, hyperinflammatory responses result in tissue damage, disruption of the endothelial barrier, and uncontrolled activation of coagulation.^54^ Overall, these findings are consistent with a model in which SARS-CoV and SARS-CoV-2 directly infect endothelial and epithelial cells, increasing levels of proinflammatory cytokines, causing immune-mediated damage to the vasculature and surrounding tissue, with exposure of tissue factor and associated thromboinflammatory changes.^91^ While these changes appear to be predominantly in the lungs, endotheliitis in COVID-19 has been observed in kidneys, liver, heart, and intestine.^91^

Additional studies in SARS-CoV and influenza found dysregulation of urokinase, coagulation, and fibrinolysis pathways contributed to the severity of lung injury, possibly through altering the hemostatic balance with subsequent coagulation-induced ischemic injury.^92^ Plasminogen was protective against severe influenza A, H5N1, and H1N1 infections.^93^ These groups hypothesized that increased fibrinolysis led to a positive feedback loop of vascular permeability, leukocyte recruitment, and fibrin generation. Interestingly, one hypothesis suggests that elevated plasminogen may be a risk factor for SARS-CoV-2 infection because plasmin may cleave the S protein of the virus and increase its infectivity.^94^ These findings highlight the delicate balance between corralling infection and uncontrolled inflammation and thrombosis.

## Therapeutic considerations

Markers of hypercoagulability and higher inflammatory mediators are consistently associated with worse outcomes in patients with ARDS and sepsis. These observations have led to numerous clinical trials targeting various components of inflammatory and coagulation pathways in acute lung injury, ARDS or sepsis. Studies with heparin, steroids, non-steroidal anti-inflammatory drugs, and TNF-α inhibitors have been disappointing.^95–100^

Given the laboratory and clinical findings in patients with severe COVID-19, several repurposed and novel therapies are under investigation in clinical trials to prevent the hyperinflammatory response or mitigate uncontrolled coagulation. As elevations in D-dimer and FDPs likely reflect ongoing lung injury and microvascular thrombi, possible therapeutic targets include inflammatory cytokines, activated platelets, neutrophils, or microparticles that may propagate thrombosis; or anticoagulants and fibrinolytics that could limit thrombosis. Supporting this enthusiasm was a recent retrospective study in China in which VTE prophylaxic dose heparin was associated with a survival benefit in patients with severe COVID-19 and evidence of sepsis-induced coagulopathy.^101^ The study found no benefit among patients with milder COVID-19 illness; however, the study did not control for other markers of disease severity nor other therapies, such as antivirals. The study raises the possibility that prophylactic or therapeutic anticoagulation may benefit patients with severe infection. Heparin may alter the biology of the disease not only through its anticoagulant properties, but also due to its anti-inflammatory effects that promote a quiescent endothelium.

Current expert recommendations, including interim guidelines from the International Society on Thrombosis and Haemostasis (ISTH) and the American College of Cardiology (ACC), recommend use of prophylactic dose LMWH or unfractionated heparin in all COVID-19 patients requiring hospital admission; for patients with a contraindication to pharmacologic prophylaxis, mechanical prophylaxis should be used.^102,103^ While a number of VTE risk stratification tools exist for hospitalized medical patients, these have not been validated in patients with COVID-19. Extended VTE prophylaxis with LMWH or direct oral anticoagulants after hospitalization for acute medical illness reduces the risk of VTE with an associated increased risk of bleeding.^104–106^ There are currently no data regarding extended prophylaxis in patients with COVID-19; however, the ACC expert opinion statement recommends consideration of extended prophylaxis in patients with elevated risk of VTE, such as patients with cancer or prolonged immobility who have low bleeding risk. Given early reports and ongoing concerns of high rates of VTE, randomized trials of empiric therapeutic anticoagulation or antifibrinolytics are ongoing, and there are reports of empiric therapeutic anticoagulation in patients with significantly elevated D-dimer both in Italy and in the US. While heparin offers both anti-inflammatory and anticoagulant effects, the benefit of therapeutic anticoagulation remains uncertain, with a risk of bleeding complications in critically ill patients with respiratory failure.^95,107^ Clinical trials will help define the role of heparin in the treatment of hospitalized patients with COVID-19. Outside of a trial setting, we advocate universal standard-dose pharmacologic VTE prophylaxis in patients without a contraindication. In patients with a high suspicion of VTE where access to confirmatory or serial imaging is limited, clinicians may consider empiric anticoagulation, although there is a paucity of evidence to provide guidance in this context. There are currently no randomized data to recommend empiric therapeutic or intermediate-dose anticoagulation in patients without documented VTE, or an other indication for anticoagulation, or outside the context of a clinical trial. A recent retrospective, observational study in New York City showed therapeutic anticoagulation was associated with decreased mortality in patients with COVID-19 who required mechanical ventilation, but not in all hospitalized patients with COVID-19. Although these findings are provocative, interpretation is limited by their observational nature.^108^

There are over 300 trials ongoing for patients with COVID-19, many of which aim to simultaneously reduce inflammation and thrombosis, including cytokine-directed therapies (against IL-1, IL-6, interferon gamma), corticosteroids, Janus kinase inhibitors, TLR ligands, complement inhibitors, *N*-acetylcysteine, serine protease inhibitors, DNAse enzymes, and anti-viral agents. However, suppressing the cytokine storm or hypercoagulability may be insufficient once initiated, and targeting upstream pathways to prevent activation of this self-amplifying feedback loop may be more effective.

One therapeutic candidate to treat COVID-19 is dipyridamole, an adenosinergic drug indicated for use as an arterial thromboembolic prophylaxis agent in combination with aspirin or warfarin.^109^ Dipyridamole has recently been shown to suppress human neutrophil and T-cell activation, upstream of cytokine effectors.^58,110^ Dipyridamole induces a type I interferon response, which is necessary for physiologic anti-viral activity, and inhibits SARS-CoV-2 replication *in vitro* by inhibiting a critical viral replication complex.^111,112^ Administered orally, dipyridamole has a favorable safety profile, and a small clinical trial in patients with COVID-19 suggests it may improve D-dimer levels.^113^ Randomized clinical trials of agents active at the intersection of inflammation and coagulation in COVID-19, such as dipyridamole, t-PA, and heparin are necessary to determine if these therapeutics can restore the balance of inflammation and coagulation without dampening early or late physiologic anti-viral responses. The heterogenous response to the SARS-CoV-2 infection and the various time-dependent pathways driving pathology make universal therapies challenging. The temporal and mechanistic role each pathway plays in severe SARS-CoV-2 infection remains uncertain and requires further exploration for treatment opportunities as efforts to control this pandemic continue.

## Conclusions

In conclusion, in patients with COVID-19, the presence of coagulopathy, characterized by elevations in D-dimer and FDPs, is consistently associated with more severe illness and mortality. Laboratory, clinical, and early histopathologic findings suggest this coagulopathy is distinct from sepsis-induced DIC and may reflect dysregulated hemostasis. Similar findings have been associated with several other viral infections, and it remains uncertain if this coagulopathy is specific to SARS-CoV-2 or the end common pathway of the thrombo-inflammatory response to severe viral infections. There are efforts to target numerous components of the thrombo-inflammatory pathway to improve outcomes in patients with severe COVID-19. The optimal management for these patients including strategies to diagnose VTE, appropriate anticoagulation doses and duration, and effectiveness of novel therapies are under active investigation in the current pandemic.
